# Systemic Factors During Metabolic Disease Progression Contribute to the Functional Decline of Adipose Tissue-Derived Mesenchymal Stem Cells in Reproductive Aged Females

**DOI:** 10.3389/fphys.2018.01812

**Published:** 2018-12-18

**Authors:** Ascentia M. Seboko, M. M. Conradie, M. J. Kruger, William Frank Ferris, Magda Conradie, Mari van de Vyver

**Affiliations:** Division of Endocrinology, Department of Medicine, Faculty of Medicine and Health Sciences, Stellenbosch University, Cape Town, South Africa

**Keywords:** interleukin 6, obesity, mesenchymal stem cells, adipose tissue, cytokines, osteogenesis, adipogenesis, body composition

## Abstract

It is known that advanced metabolic disorders such as type 2 diabetes compromise the functional and regenerative capacity of endogenous adipose-tissue resident stem cells (ADSCs). It is, however, still unclear at which stage of disease progression ADSCs become compromised and whether systemic factors contribute to their functional decline. It was therefore hypothesized that inflammatory changes in the systemic microenvironment during distinct stages of disease progression negatively affect the functional capacity of ADSCs. A total of forty-seven (*n* = 47) black African reproductive aged females (32 ± 8 years; mean ± SD) were included in this study and subdivided into: (a) healthy lean (C; body mass index, BMI ≤ 25 kg/m^2^), (b) healthy overweight/obese (OB; BMI ≥ 25 kg/m^2^), (c) obese metabolic syndrome (MetS; BMI ≥ 30 kg/m^2^), and (d) type 2 diabetes mellitus (T2DM; previously diagnosed and on treatment) groups. Participants underwent anthropometric assessments and a DXA scan to determine their body composition and adipose indices. Each persons’ systemic metabolic- (cholesterol, HDL, LDL, triglycerides, and blood glucose) and inflammatory profiles (CRP, SDF1α, TNFα, IL6, IL8, IL10, and IFNy) were also evaluated. Participant-derived serum was then used to treat an ADSC cell line *in vitro* and its effect on viability (MTT-based assay), proliferation (BrdU), migration (wound healing assay), and osteogenic differentiation assessed. When exposed to serum derived from overweight/obese individuals (with or without metabolic syndrome), both the proliferative and migratory responses of ADSCs were less pronounced than when exposed to healthy control serum. Serum IL6 concentrations were identified as a factor influencing the proliferation of ADSCs, suggesting that long-term disruption to the systemic cytokine balance can potentially disrupt the proliferative responses of ADSCs. Obese participant-derived serum (with and without metabolic syndrome) furthermore resulted in lipid accumulation during osteogenic differentiation. This study, therefore demonstrated that systemic factors in obese individuals, regardless of the presence of metabolic syndrome, can be detrimental to the multifunctional properties of ADSCs.

## Introduction

The dynamic shift to urbanization has had various implications on the lives of people globally, with an astounding increase in the prevalence of obesity (James et al., 2004; Kelly et al., 2008). According to the WHO, in 2014 over 600 million people worldwide were considered obese (WHO, 2018) and it is predicted that by the year 2030, approximately 1 billion people will be affected by this lifestyle related disorder (Kelly et al., 2008). This is especially evident in the female ethnic populations of developing countries such as South Africa. Predictions indicate that currently 70% of South African females are overweight and 40% are at risk for developing obesity-associated health problems (Sartorius et al., 2015).

Disruption of the normal endocrine system function during obesity affects glucose homeostasis and causes disease progression toward metabolic syndrome (visceral adiposity with dyslipidemia) and potentially the development of T2DM (Martyniak and Masternak, 2017; Pirola and Ferraz, 2017). Bone marrow mesenchymal stem cell dysfunction is a known complication of type 2 diabetes and occurs because of pathological changes within the stem cell niche microenvironment (van de Vyver et al., 2016; van de Vyver, 2017). Similarly, the microenvironment within adipose tissue has also been shown to modulate the proliferation, migration, and immunophenotypic profile of ADSCs (Pachón-Peña et al., 2016).

Changes in the adipose tissue cellular composition during the development of obesity is considered a major cause of metabolic dysregulation and disease progression (Martyniak and Masternak, 2017). Adipocyte hypertrophy, chronic adipose tissue inflammation, and the formation of crown-like structures (excessive M1 proinflammatory macrophage infiltration) contribute to the development of insulin resistance and is associated with a lack of adipocyte turnover (Barbagallo et al., 2017; Pirola and Ferraz, 2017). The excessive infiltration of M1 macrophages into adipose tissue has also been shown to limit the self-renewal capacity of ADSCs (Martyniak and Masternak, 2017) and it is known that ADSCs derived from diabetic patients often fail to differentiate into functional adipocytes *in vitro* (Barbagallo et al., 2017). Taken together these studies, suggest that inflammatory changes within the localized tissue microenvironment can impair the function of ADSCs. It is, however, unclear at which stage of disease progression ADSC function becomes compromised, since adipose tissue inflammation is also thought to play a key role in governing the plasticity of ADSCs (Strong et al., 2015; Badimon and Cubedo, 2017). Furthermore, it is unknown whether systemic factors contribute to the functional decline of ADSCs or if it is solely due to changes in the localized tissue environment.

We hypothesize that inflammatory changes in the systemic/circulating microenvironment during disease progression contribute to the functional decline of ADSCs by negatively affecting their growth, mobility and differentiation capacity. To test this hypothesis, the inflammatory and metabolic profile of healthy lean, healthy overweight/obese, obese with metabolic syndrome, and type 2 diabetic reproductive aged females were determined. Participant-derived serum was then used to treat a healthy ADSC cell line *in vitro* and subsequent changes in the functional capacity of ADSCs assessed.

## Materials and Methods

This study was approved by the health research ethics committee at Stellenbosch University (N15/07/066) and permission was obtained for the research to be conducted at Tygerberg Academic Hospital from the Western Cape, Department of Health (WC_2016RP14858). Volunteers were informed of the purpose and risks of the study before signing an informed consent form. All experimental procedures were conducted according to the ethical guidelines and principles of the declaration of Helsinki.

### Participant Recruitment and Sample Collection

A total of forty-seven (*n* = 47) black African reproductive aged females (32 ± 2 years; mean ± SE) were included in this study and were subdivided into: (a) healthy lean (C; *n* = 8; BMI ≤ 25 kg/m^2^), (b) healthy obese (OB; BMI ≥ 25 kg/m^2^, < 2 metabolic risk factors; *n* = 25), (c) obese metabolic syndrome (MetS; BMI ≥ 30 kg/m^2^,≥3 metabolic risk factors; *n* = 9), and (d) T2DM (*n* = 5; previous diagnosis, on treatment) groups. Refer to Table 1 for normal physiological ranges of metabolic risk factors, the criteria used for identifying participants with metabolic syndrome complied with the recommendations of the International Diabetes Federation (IDF) (International Diabetes Federation, 2005).

**Table 1:** Criteria for grouping of participants.

	C	OB	MetS	T2DM
**BMI** (kg/m^2^)	≤25	>25	>30	>30
**Visceral adiposity:**	No	No	Yes	
Waist-to-hip ratio ≥ 0.85				
Trunk-to-limb fat mass ratio (TF/LF):>1				
**Metabolic risk factors:**	<2	<2	≥2	Previous
TGS ≥ 1.7 mmol/L				diagnosis
HDL < 1.29 mmol/L				
BP systolic ≥ 130 mmHg; diastolic ≥ 85 mmHg				
FBG ≥ 5.6 mmol/L				

*Healthy lean participants (C) had a normal BMI of 20–25 kg/m^*2*^, no visceral adiposity and less than two metabolic risk factors. Overweight/obese (OB) participants had a BMI of above 25 kg/m^*2*^, no visceral adiposity, and less than two metabolic risk factors. Metabolic syndrome (MetS) participants had a BMI of above 30 kg/m^*2*^, visceral adiposity, and more than two metabolic risk factors. Type 2 diabetes mellitus (T2DM) participants were all previously diagnosed and on treatment. BP, blood pressure, FBG, fasting blood glucose, HDL, high density lipoprotein, TGS, triglycerides*.

All participants completed nutritional and lifestyle questionnaires, underwent a DXA scan (body composition and bone density; Hologic Discovery W, Serial Number 70215, Software Version 13.1, Hologic Inc., Marlborough, MA, United States) and anthropometrical measurements [height (m), weight (kg), hip circumference (cm), waist circumference (cm)]. Fasting blood glucose levels (finger prick) were determined using the Contour plus glucometer system (BAYER, Leverkusen, Germany) and whole blood samples were collected into EDTA and serum separating (SST) tubes (BD Vacutainer, Franklin Lakes, NJ, United States).

#### Serum Sample Processing and Analysis

Whole blood collected in the SST tubes (BD Vacutainer, Franklin Lakes, NJ, United States) were allowed to clot at room temperature before centrifugation for 10 min @ 3000 RPM, 4°C. Serum was aliquoted and stored at -80°C until subsequent analysis for metabolic (lipid profile) and inflammatory (cytokine) markers. Each participant’s total cholesterol, TGS, HDL, and LDL were determined using an Alere Afinion Lipid^TM^ Panel (#10183107, Alere, Waltham, MA, United States) in combination with the Afinion AS100 Analyzer (Alere, Waltham, MA, United States). Serum cytokine concentrations were quantified using either a Bio-Plex Pro Human Cytokine assay kit [interleukin 8 (IL8), IFNγ, and tumor necrosis factor alpha (TNFα); Bio-Rad, Hercules, CA, United States] or human ELISA kits targeting CRP (ab99995, Abcam, Cambridge, United Kingdom), SDF1α (#0184080916, Thermo Fisher Scientific, Waltham, MA, United States), interleukin 6 (IL6; #B228198, BioLegend, San Diego, CA, United States), and interleukin 10 (IL10; #B238108, BioLegend, San Diego, CA, United States).

#### PBMC Isolation and Analysis

Peripheral blood mononuclear cells were isolated from the whole blood collected in EDTA tubes using density centrifugation. Whole blood was layered upon an equal volume of Histopaque-1077 (Sigma-Aldrich, St. Louis, MO, United States) and centrifuged at 400× *g* for 30 min at room temperature. The opaque interface containing PBMCs were transferred to a new tube, washed using PBS (centrifuged 250× *g*, 10 min) and resuspended into *freezing media* before storage at -80°C. Freezing media consisted of 20% HSA (Sigma-Aldrich, St. Louis, MO, United States) and 10% DMSO (Merck, Darmstadt, Germany) in DMEM with ultra-glutamine (BioWhittaker, Lonza, Basel, Switzerland). The percentage of circulating progenitor/stem cells within the PBMC population was determined using flow cytometry (FACSCanto II flow cytometer with FACSDiva software, BD Biosciences, San Jose, CA, United States). PBMCs at a concentration of 1 × 10^6^ cells/mL were co-labeled with a cocktail of fluorochrome-conjugated monoclonal antibodies: FITC mouse anti-human CD45 (555482, BD Pharmingen, BD Biosciences, CA, United States) and APC mouse anti-human CD34 (555824, BD Pharmingen, BD Biosciences, CA, United States). Since a multi-color cytometric analysis was performed, fluorescent compensation settings were established through a compensation experiment (Comp beads, BD Biosciences, CA, United States) and regions of positive and negative staining were determined through a FMO experiment. Data analysis was performed using FlowJo Vx (Treestar, Woodburn, OR, United States) software.

### *In vitro* Cell Culture Experiments

#### Standard Cell Culture Conditions

A human adipose tissue-derived mesenchymal stem cell line (ADSCs; Donor 26508, #000034977, Poietics, Lonza, Basel, Switzerland) was maintained at 37°C, in 90% humidified air with 5% CO_2_. SGM consisted of high-glucose (4.5 g/L) DMEM with ultra-glutamine (BioWhittaker, Lonza, Basel, Switzerland), 1% penicillin/streptomycin (BioWhittaker, Lonza, Basel, Switzerland), and 10% FBS (Biochrom, Berlin, Germany). For culture expansion, adherent ADSCs were liberated with 0.5% trypsin (200 mg/L Versene EDTA, #BE171616, Lonza, Belgium) and sub-cultured at a seeding density of 5000 cells/cm^2^. For cryopreservation, ADSCs were stored in liquid N_2_ in SGM containing 10% DMSO (Merck, Darmstadt, Germany). All experiments were performed in triplicate on ADSCs between passages five and seven.

#### Cellular Proliferation and Viability Assays

The effect of participant-derived serum (*n* = 47) on ADSC viability and proliferation was investigated by replacing the FBS in the SGM with serum derived from participants. Patient-derived serum was collected and stored as described in the section “Serum Sample Processing and Analysis.” For the *in vitro* viability and proliferation assays, cells were treated with media consisting of high-glucose (4.5 g/L) DMEM with ultra-glutamine (BioWhittaker, Lonza, Basel, Switzerland), 1% penicillin/streptomycin (BioWhittaker, Lonza, Basel, Switzerland), and 20% serum derived from a single participant. It should be noted that serum derived from participants were not pooled for the proliferation and viability assays, and the *in vitro* experiments were thus repeated in duplicate for each individual participant (*n* = 47). Cell viability was assessed after 24 h of exposure to participant-derived serum using an MTT (3-(4,5-dimethylthiazolyl-2)-2,5-diphenyltetrazolium bromide) based In Vitro Toxicology kit (#SLBM0752V, Sigma-Aldrich, Schnelldorf, Germany) according to the manufacturer’s instructions. Similarly, the effect of participant-derived serum on the proliferation rate of ADSCs were assessed over a period of 24 h by using a Bromodeoxyuridine (BrdU) cell proliferation ELISA kit (#11417600, Roche, Basel, Switzerland) according to the manufacturer’s instructions. To confirm the observed association between serum interleukin 6 (IL6) concentrations and cellular proliferation (refer to the Results section The Viability/Proliferation) a dose response experiment was performed; ADSCs were treated with various physiological concentrations (1, 2, 4, 8, 10, 16, 20, and 25 pg/mL) of recombinant IL6 (575706, BioLegend, San Diego, CA, Untied States) and the proliferative response of ADSCs assessed over a period of 24 h as described above (BrdU ELISA, #11417600, Roche, Basel, Switzerland).

#### *In vitro* Wound Healing Assay

The migration capacity of ADSCs and their ability to close an *in vitro* wound was assessed using an *in vitro* migration assay (Ibidi GmbH, Munich, Germany). ADSCs were seeded into both chambers of a 35 mm high u-dish with culture-insert (Ibidi GmbH, Munich, Germany) and the cells were allowed to reach confluence under standard growth conditions (SGM). Upon reaching confluence, the culture insert was removed and SGM replaced with media containing participant-derived serum. Cellular migration and the percentage closure of the cell-free gap was determined over a period of 24 h. For each assay, four random images were taken using a light microscope (Olympus CKX41, CachN 10×/0.25 PhP objective) and EOS600D Canon digital camera at 0, 4, 7, and 24 h. The cell free area was calculated by tracing along the border of the wound using Image J software (version 1.46, NIH, MD, United States) and the surface area (μm^2^) of the cell-free gap determined. The percentage gap closure (% gap closure) was calculated using the following equation: [(area 0 h-area *n* h)/(area 0 h)] × 100. It should be noted that serum derived from participants were not pooled for the migration assay, and the *in vitro* experiments were thus completed for each individual participant (*n* = 47). To confirm the observed association between serum interleukin 8 (IL8) concentrations and the migration rate of cells, a dose response experiment was performed; ADSCs were treated with physiological concentrations (1, 2, 4, 8, 10, 16, 20, and 25 pg/mL) of IL8 (I1645, Sigma-Aldrich, Merck, United States) and cellular migration assessed over a period of 24 h as described above (*In vitro* migration assay, Ibidi GmbH, Munich, Germany).

#### Multi-lineage Differentiation Experiments

The effect of participant-derived serum on the multi-lineage differentiation capacity of ADSCs were assessed using standardized adipogenic and osteogenic differentiation protocols. For adipogenic differentiation, ADSCs were exposed to AM in the presence of participant-derived serum for a period of 14 days with media being changed twice weekly. The extent of lipid accumulation was quantified using Oil Red O staining as previously described (van de Vyver et al., 2014; Jacobs et al., 2016). AM consisted of: high-glucose (4.5 g/L) DMEM with ultra-glutamine (BioWhittaker, Lonza, Basel, Switzerland), 1% penicillin/streptomycin (BioWhittaker, Lonza, Basel, Switzerland), 10 mM insulin (SLBD5980, Sigma Life Sciences), 0.5 mM 3-isobutyl-1-methylxanthine (IBMX, STBC7632V, Sigma Life Sciences), 1 mM dexamethasone (BCBK1265V, Sigma Life Sciences), 56 mM indomethacin (064K1207, Sigma Life Sciences), and 20% participant-derived serum.

For osteogenic differentiation ADSCs were exposed to OM in the presence of participant-derived serum for a period of 21 days with media being changed twice weekly. OM consisted of: high-glucose (4.5 g/L) DMEM with ultra-glutamine (BioWhittaker, Lonza, Basel, Switzerland), 1% penicillin/streptomycin (BioWhittaker, Lonza, Basel, Switzerland), 10 nM dexamethasone (BCBK1265V, Sigma Life Sciences), 50 mM ascorbic acid (SLBB4446V, Sigma Life Sciences), 10 mM β-glycerophosphate (029K54241V, Sigma Life Sciences), and 20% participant-derived serum. The extent of mineralization was quantified using Alizarin Red S staining. Note serum from all participants were pooled for both the adipogenic and osteogenic differentiation experiments.

The effect of IL6 on ADSC differentiation were assessed by adding physiological relevant concentrations (IL6 8 pg/mL) to either the standard AM or OM differentiation media (without the presence of participant-derived serum). For quantification purposes, four random images per well were taken using a light microscope (Olympus CKX41, CachN 10×/0.25 PhP objective) and EOS600D Canon digital camera. The percentage surface area stained with either Oil Red O (lipid accumulation) or Alizarin Red (mineralization) was quantified using Image J software (version 1.46, NIH, MD, United States) as previously described (van de Vyver et al., 2014; Jacobs et al., 2016).

### Statistical Analysis

All values are presented as mean ± standard error of the mean (mean ± SE). Statistical analysis was performed using Statistica software (version 13, StatSoft, Johannesburg, South Africa). One Way ANOVA with Tukey *post hoc* test was used to determine group effects. In cases where data was not normally distributed, non-parametric Kruskal–Wallis ANOVA with Dunn’s multiple comparison test was used to determine differences between groups. Factorial ANOVA with Tukey *post hoc* test was used to determine the effect of group × time. To account for the age difference between groups, analysis of co-variance (ANCOVA) was performed with age set as confounding factor. Case-wise Product–Moment correlation analysis was used to determine associations between serum cytokine concentrations and changes in the functional capacity of ADSCs. Level of significance was accepted at *p* < 0.05.

## Results

### Participant Characteristics

#### Body Composition and Metabolic Profile of Study Participants

**FIGURE 1:**
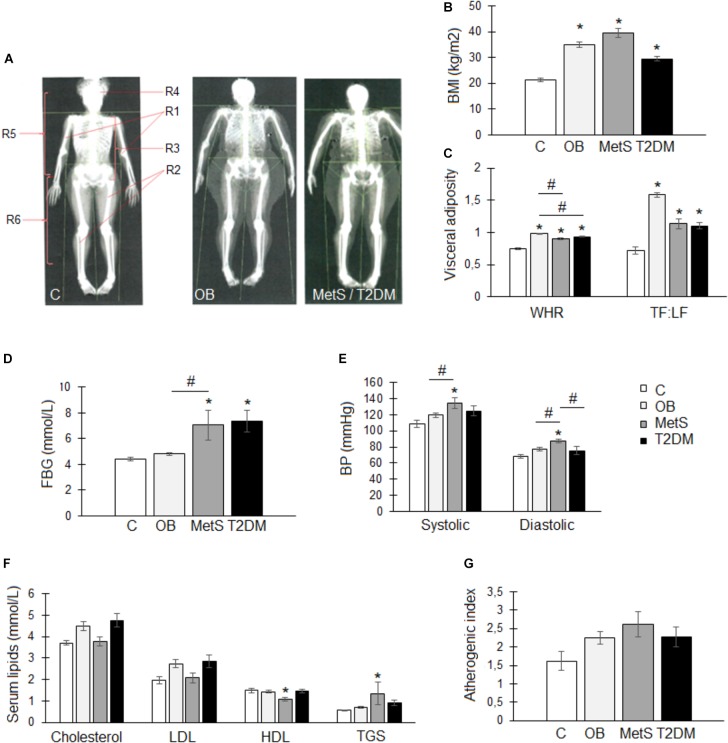
Body composition and metabolic profile of participants within the healthy lean (C), overweight/obese (OB), metabolic syndrome (MetS), and type 2 diabetic (T2DM) groups. **(A)** Representative images of whole body DXA scans illustrating a normal (C), gynoid-shaped (OB), and android-shaped (MetS/T2DM) body composition. Body regions: R1, arms, R2, legs, R3, android, R4, head, R5, trunk, and R6, gynoid. **(B)** Body mass index (BMI; kg/m^2^). **(C)** Visceral adiposity measures: waist-to-hip ratio (WHR) and trunk-to-limb fat mass ratio (TF:LF). **(D)** Fasting blood glucose (FBG) levels (mmol/L). **(E)** Systolic and diastolic blood pressure (BP; mmHg). **(F)** Serum lipid levels: total cholesterol (mmol/L), low density lipoprotein (LDL; mmol/L), high density lipoprotein (HDL; mmol/L), and triglycerides (TGS; mmol/L). **(G)** Atherogenic index was calculated using the following formula: (total cholesterol–HDL)/HDL. Statistical analysis: analysis of co-variance (confounding factor: age) with Tukey *post hoc* test. ^∗^*p* < 0.05 indicates significant difference from healthy lean (C) group. ^#^*p* < 0.05 indicates significant difference between groups.

All participants had similar lifestyle and nutritional habits. The healthy lean participants were, however, younger (*p* < 0.05) than participants from the other groups (Table 2) and were the only group with a normal body composition (35 ± 2% body fat; BMI 21 ± 1 kg/m^2^) (Figures 1A,B). OB participants had a gynoid body shape (49 ± 1% body fat; BMI 35 ± 1 kg/m^2^) with fat distributed predominantly in the subcutaneous regions, whereas the MetS (48 ± 2% body fat; BMI 40 ± 2 kg/m^2^) and T2DM (39 ± 2% body fat; BMI 29 ± 1 kg/m^2^) participants had visceral adiposity (android-shaped body; *p* < 0.05) (Figures 1A–C). Although OB and MetS participants had a higher BMI (*p* < 0.05), WHR (*p* < 0.05), and TF/LF ratio (*p* < 0.05) compared to the healthy lean participants (Figures 1B,C), visceral adiposity (WHR > 0.85 and TF/LF > 1) and hyperglycemia (FBG > 5.6 mmol/L) were only evident in the MetS and T2DM groups (Figures 1C,D). Furthermore, the MetS participants suffered from hypertension (systolic > 130 mmHg, diastolic > 85 mmHg) and dyslipidemia (HDL < 1.29 mmol/L and TGS levels > 1.3 mmol/L) (Figures 1E–G), suggesting that in this population with similar lifestyle and nutritional habits, disease progression occurs with age and is dependent on body composition. To correct for the age difference between groups, ANCOVA was performed for all subsequent data analysis.

**Table 2:** Lifestyle and nutritional information.

	C	OB	MetS	T2DM
Age (years)	26 ± 1	31 ± 2	35 ± 3	41 ± 1
BMI (kg/m^2^)	21 ± 1	35 ± 1	39 ± 2	29 ± 1
**Lifestyle**				
Physical activity (Borg scale)	2.4 ± 0.4	2.4 ± 0.2	2.8 ± 0.2	2.6 ± 0.4
Sleeping (>6 h per night)	87%	76%	100%	80%
Smoking	37%	12%	0%	0%
Alcohol	62%	40%	22%	20%
**Nutrition**				
Full meals (per day)	2.6 ± 0.2	2.4 ± 1.4	2.6 ± 0.2	2.4 ± 0.2
Snacks (per day)	1.8 ± 0.4	1.2 ± 0.2	0.5 ± 0.4	1.6 ± 0.5
Fruits (per week)	6 ± 1	6 ± 1	5 ± 1	6 ± 2
**Adipose indices**				
Total body fat (%)	35 ± 2	49 ± 1^∗^	48 ± 2^∗^	39 ± 2^∗^
Android: Gynoid fat mass (ratio)	0.76 ± 0.04	0.98 ± 0.01	1.02 ± 0.03^∗^	1.01 ± 0.05^∗^
**Medication**	N/A	N/A	Hypertension	Metformin
				Insulin

*Values are presented as mean ± SE. Physical activity scale: (1) sedentary; (2) light activity (no increase in heart rate); (3) moderate activity (10 min/day); (4) vigorous activity (>1 h per day on most days). Statistical analysis: one-way ANOVA with Tukey *post hoc* test. ^∗^*p* < 0.05 indicate significant difference from healthy lean (C) group*.

#### Systemic Inflammatory Profile of Study Participants

For each individual participant serum pro- and anti-inflammatory cytokines were assessed as indication of their current inflammatory status.

**FIGURE 2:**
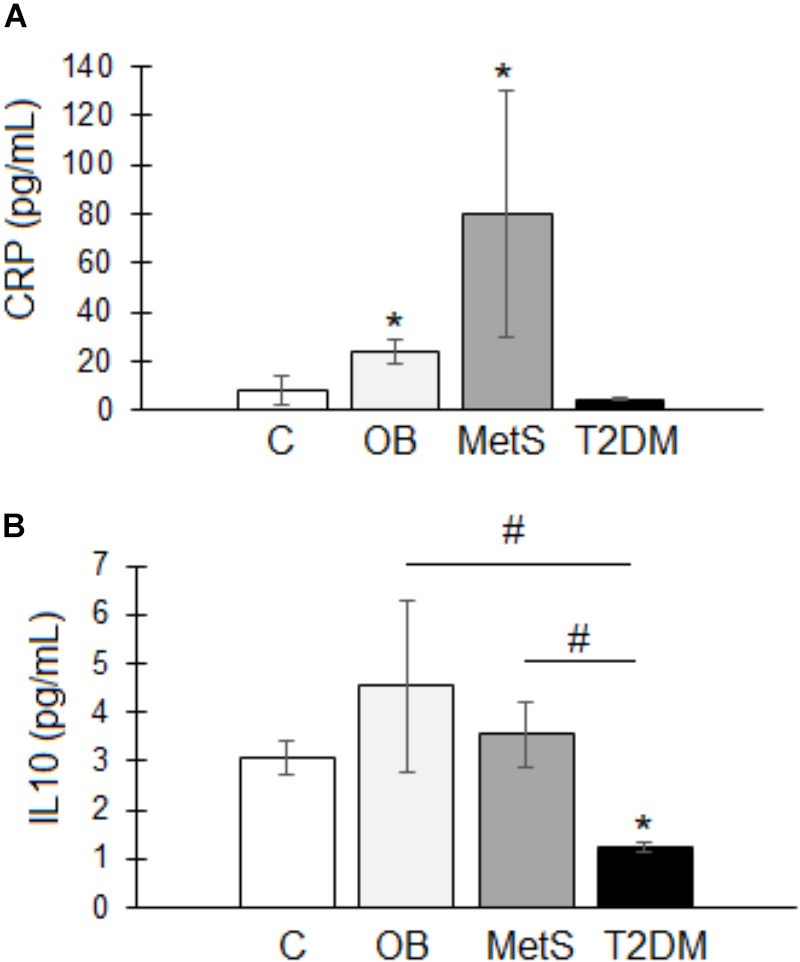
Serum inflammatory profile of participants within the healthy lean (C), overweight/obese (OB), metabolic syndrome (MetS), and type 2 diabetic (T2DM) groups. **(A)** Pro-inflammatory C-Reactive protein (CRP; pg/mL). **(B)** Anti-inflammatory interleukin 10 (IL10; pg/mL). Statistical analysis: non-parametric Dunn’s multiple comparisons test with Kruskal–Wallis *post hoc* test. ^∗^*p* < 0.05 indicates significant difference from healthy lean (C) group. ^#^*p* < 0.05 indicates significant difference between groups.

Pro-inflammatory parameters: systemic inflammation was evident in the OB and MetS groups, with CRP levels (OB 24 ± 5 and MetS 80 ± 50 pg/mL) significantly elevated (*p* < 0.05) compared to healthy lean (8 ± 6 pg/mL) participants (Figure 2A). No difference was, however, detected in serum pro-inflammatory TNFα- (C 4.8 ± 1; OB 4.9 ± 2; MetS 4.7 ± 0.8; and T2DM 3.8 ± 0.3 pg/mL) or IFNγ (C 1 ± 0.1; OB 1 ± 0.1; MetS 0.9 ± 0.1; and T2DM 1.1 ± 0.1 pg/mL) levels between groups.

Anti-inflammatory parameters: IL10 was significantly reduced in T2DM (1.2 ± 0.1 pg/mL) participants compared to the other groups (C 3 ± 0.4; OB 4.5 ± 1.7; and MetS 3.5 ± 0.6 pg/mL) (Figure 2B). Note: the T2DM group were on treatment (Insulin/Metformin or a combination thereof) (Table 2) and their metabolic parameters relatively well controlled.

#### Circulating Stem/Progenitor Cells

**FIGURE 3:**
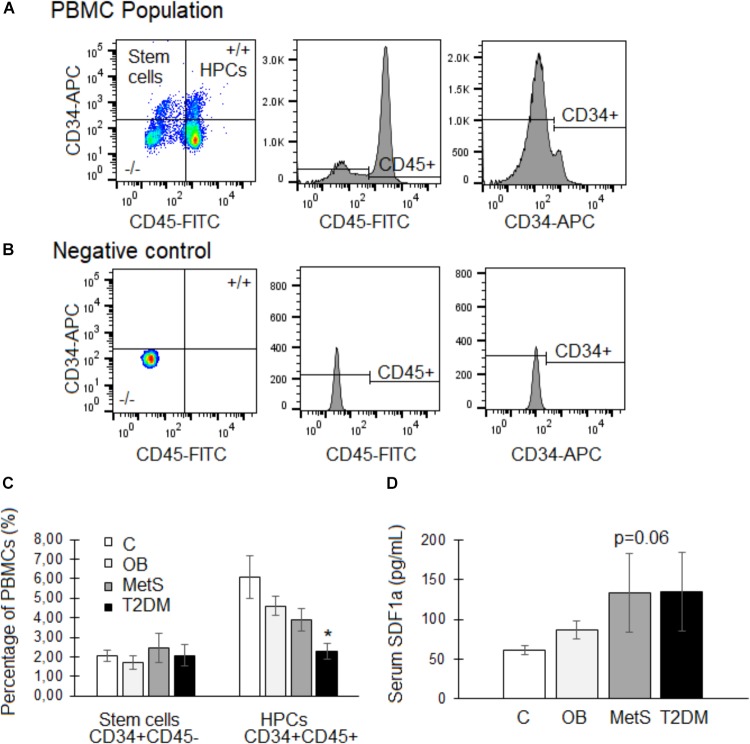
Circulating stem/progenitor cells and serum SDF1α levels of participants within the healthy lean (C), overweight/obese (OB), metabolic syndrome (MetS), and type 2 diabetic (T2DM) groups. **(A,B)** Representative flow cytometry plots of CD34 (APC conjugated) vs. CD45 (FITC conjugated) expression within the peripheral blood mononuclear cell (PBMC) population. Hematopoietic progenitor cells (HPCs) express both the stem cell marker CD34 as well as the hematopoietic lineage marker CD45, whereas stem cells do not express CD45. **(C)** Quantification of the percentage of stem cells (CD34 + CD45–) and HPCs (CD34 + CD45+) within the PBMC population. **(D)** Serum levels (pg/mL) of the chemokine, SDF1α. Statistical analysis: non-parametric Dunn’s multiple comparisons test with Kruskal–Wallis *post hoc* test. ^∗^*p* < 0.05 indicates significant difference from healthy lean (C) group.

Despite a slight tendency (*p* = 0.06) toward higher serum levels of the chemokine SDF1α in the MetS and T2DM groups compared to the healthy lean group, a significant reduction was evident in the percentage of circulating HSCs within these two groups (Figure 3). With disease progression the percentage of HSCs declined from 6.1 ± 0.8% in the healthy lean group (C) to 4.6 ± 0.5% in the OB, 3.9 ± 0.6% in the MetS, and 2.3 ± 0.4% in the T2DM (*p* < 0.05) group (Figure 3C).

### The Effect of Participant-Derived Serum on ADSC Functional Abilities

#### The Viability/Proliferation

**FIGURE 4:**
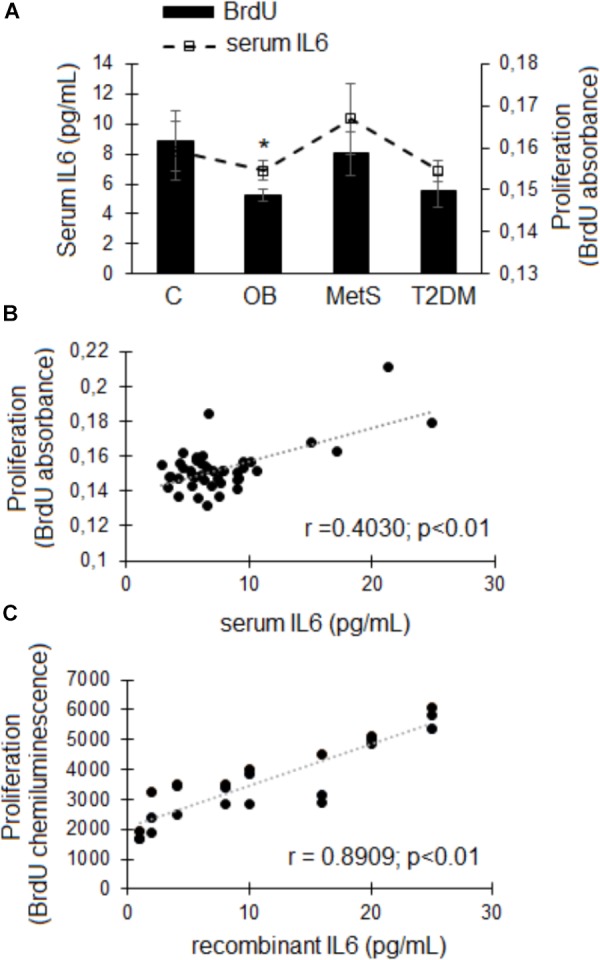
The effect of participant-derived serum on ADSC proliferation. **(A)** Cellular proliferation (bars) and serum IL6 concentrations (pg/mL; dotted line). Statistical analysis: non-parametric Dunn’s multiple comparisons test with Kruskal–Wallis *post hoc* test. ^∗^*p* < 0.05 indicates significant difference from healthy lean (C) group. **(B)** Correlation between the IL6 concentrations in participant-derived serum and ADSC proliferative responses over a period of 24 h. **(C)** Correlation between increasing physiologically relevant concentrations of recombinant IL6 and ADSC proliferation (*n* = 3). Statistical analysis: Spearman’s ranked correlation analysis with two-tailed *p*-value. Level of significance accepted at *p* < 0.05.

Replacement of FBS with participant-derived serum did not affect the viability of ADSCs. An *in vitro* MTT-based toxicology assay demonstrated that participant-derived serum (all groups) could sustain these ADSCs in culture to a similar extent as standard culture conditions with FBS over a period of 24 h (data not shown). A significant correlation (*r* = 0.4030, *r*^2^ = 0.1624, *p* < 0.01; *n* = 47) was evident between the concentration of IL6 in participant-derived serum (C 8.2 ± 1.9; OB 6.8 ± 0.6; MetS 10.3 ± 2.4; and T2DM 6.9 ± 0.7 pg/mL) and the proliferative response of ADSCs (Figures 4A,B). This was confirmed using a recombinant IL6 dose response experiment (*n* = 3), demonstrating that an increase in the rate of cellular proliferation (BrdU incorporation) corresponds with increasing concentrations of rhIL6 (*r* = 0.8909, *r*^2^ = 0.7936, *p* < 0.01) (Figure 4C).

#### Wound/Migration

**FIGURE 5:**
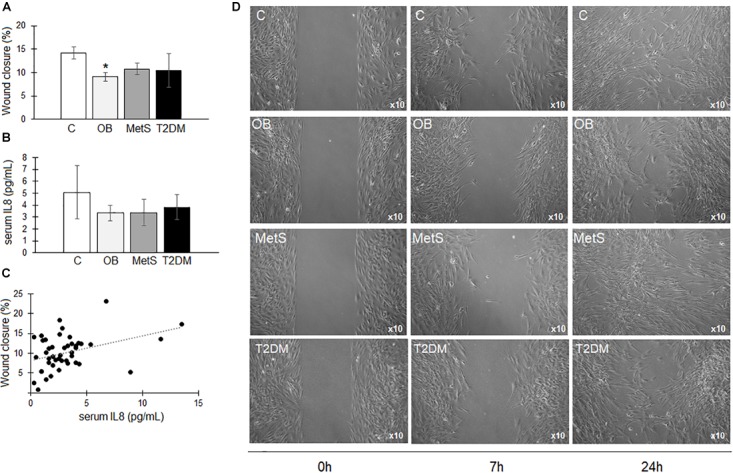
The effect of participant-derived serum on ADSC migration. **(A)** The percentage wound closure over a period of 7 h in the presence of serum derived from participants within the healthy lean (C), overweight/obese (OB), metabolic syndrome (MetS), and type 2 diabetic (T2DM) groups. **(B)** IL8 concentrations (pg/mL) in participants’ serum. **(C)** Non-significant correlation between the IL8 concentrations in participant-derived serum and ADSC proliferative responses over a period of 24 h. **(D)** Representative images of ADSC migration at 0, 7, and 24 h in the presence of participant-derived serum. Statistical analysis: non-parametric Dunn’s multiple comparisons test with Kruskal–Wallis *post hoc* test. ^∗^*p* < 0.05 indicates significant difference from healthy lean (C) group.

There was a significant reduction (*p* < 0.05) in the migration rate of ADSCs over a period of 7 h in the presence of OB participant-derived serum compared to the C group (Figure 5A). Even though a possible association (*r* = 0.5602, *p* < 0.05) was evident between serum IL8 concentrations (C 5 ± 2; OB 3 ± 1; MetS 3 ± 1; and T2DM 4 ± 1 pg/mL) and the migration rate of ADSCs (Figures 5B,C), this could not be confirmed in a dose response experiment. Representative images of ADSC migration is presented in Figure 5D.

#### Adipogenic and Osteogenic Differentiation

**FIGURE 6:**
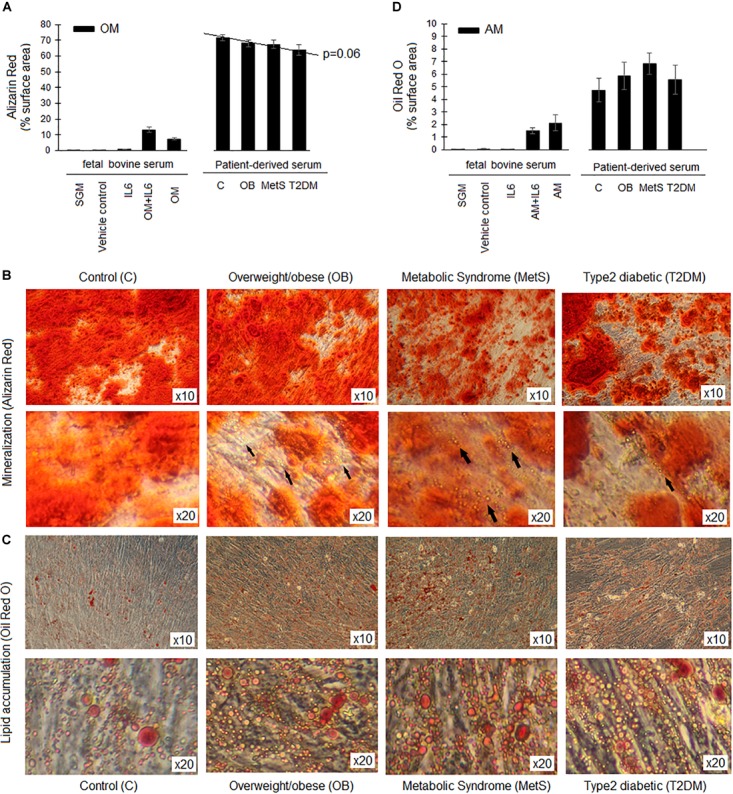
Differentiation capacity of ADSCs in presence of participant-derived sera. **(A)** The extent of mineralization (Alizarin Red staining) during osteogenesis (OM) over a period of 21 days. **(B)** Representative images of Alizarin Red S staining after 21 days of osteogenesis in the presence of sera derived from either healthy lean (C), overweight/obese (OB), metabolic syndrome (MetS), or type 2 diabetic (T2DM) participants. Arrows indicate the presence of lipid droplets within the mineralized matrix following osteogenesis. **(C)** Representative images of Oil Red O staining after 14 days of adipogenesis in the presence of sera derived from either C, OB, MetS, or T2DM participants. **(D)** The extent of lipid accumulation (Oil Red O staining) during adipogenesis (AM) over a period of 14 days. Statistical analysis: one-way ANOVA with Tukey *post hoc* test.

The presence of participant-derived serum in the differentiation media promoted the differentiation of ADSCs to a greater extent than FBS (Figures 6A,D). A small non-significant tendency toward reduced osteogenic differentiation and increased adipogenesis was observed in the presence of serum-derived from participants at different stages of metabolic disease (Figure 6). At higher magnification, lipid droplets were evident in the mineralized areas following osteogenesis in the presence of serum derived from obese (OB, MetS, and T2DM) participants. These lipid droplets were, however, not evident following osteogenesis in the ADSCs treated with serum derived from the healthy lean participants (Figures 6A,B). In contrast, a slight non-significant increase in adipogenesis was observed in the presence of serum derived from participants in the OB and MetS groups (Figures 6A,C). Refer the section “Multi-lineage Differentiation Experiments” for detailed information on the differentiation protocols used.

## Discussion

Autologous adipose tissue-derived stem cell therapy for the treatment of numerous secondary complications associated with obesity-induced type 2 diabetes (retinopathy, neuropathy, and non-healing foot ulcers) has become the focus of numerous investigations (Zhang et al., 2012; Kaisang et al., 2017; Wang et al., 2017). The reason for this is because these stromal cells have a therapeutic capacity that is comparable to that of bone marrow-derived mesenchymal stem cells (Dmitrieva et al., 2012; Hsiao et al., 2012; Jin et al., 2013) and are in sufficient quantities easily attainable for therapeutic purposes (Schäffler and Büchler, 2007 Mailey et al., 2014). Even though previous studies have shown deficiencies in the functional capacity of ADSCs-derived from type 2 diabetic and metabolic syndrome patients (Oliva-Olivera et al., 2015; Pachón-Peña et al., 2016; Barbagallo et al., 2017), it is still unclear at which stage of disease progression ADSCs become compromised and whether systemic factors contribute to their functional decline. In this study, we demonstrated for the first time that systemic factors in obese individuals, regardless of dyslipidemia, affects the *in vitro* functional capacity of ADSCs. This is in agreement with the outcome of a recent systematic review indicating that there is currently no evidence to suggest that cholesterol and hypertension affect ADSC function (Varghese et al., 2017).

In the present cross-sectional study, all participants had similar lifestyle and nutritional habits (with the exception of occasional smoking and alcohol consumption) yet metabolic disturbances (such as dyslipidemia and hyperglycemia) were only evident in the older participants with an android body shape. This is consistent with the literature indicating that metabolic disease progression is dependent on body composition and occurs over time with age being an confounding factor (Giudici et al., 2017; Motamed et al., 2017). Slight elevations in serum SDF1α levels were furthermore indicative of disease progression (Jung et al., 2009) and progenitor cell mobilization defects (Jung et al., 2009; Jialal et al., 2010) in the metabolic syndrome and type 2 diabetes groups; SDF1α is known to be a potent inducer of stem cell mobilization and migration (Hattori et al., 2001; Huang and Liu, 2012), but metabolic and inflammatory dysregulation during obesity seem to have disrupted the chemotactic effects of this factor (Holmes, 2015). A steady decline in the percentage of HSCs was present within the circulating PBMC pool during obesity-associated disease progression even though SDF1α signaling was upregulated in the metabolic syndrome and type 2 diabetes groups. Taken together, with evidence of systemic inflammation (elevated CRP levels within the OB and MetS), the current grouping of participants provides a model for assessing the influence of systemic factors at distinct stages of metabolic disease on ADSC function. Future studies should, however, aim to identify novel cytokines/factors in patients that contribute to the functional decline of ADSCs as this was beyond the scope of this study.

The multi-lineage differentiation experiments in the presence of participant-derived serum that is representative of various disease stages indicated that systemic factors during obesity compromises the regenerative potential of ADSCs. It should, however, be noted that the serum was pooled for the differentiation experiments and that gene expression analysis is needed to give more mechanistic insight into the observations of the current study. This study suggests that osteogenesis was accompanied by lipid accumulation when ADSCs were exposed to obesity-associated systemic factors. Oliva-Olivera et al. (2015) previously demonstrated a decrease in the osteogenic potential of endogenous ADSCs in individuals with metabolic syndrome and that alterations occur in the transcriptional pattern of enzymes involved in cellular redox balance, suggesting that slight changes within the gene expression profile of endogenous ADSCs may alter their therapeutic efficacy.

When exposed to serum derived from overweight/obese individuals, both the proliferative and migratory response of ADSCs were furthermore less pronounced than when exposed to healthy control serum. This corresponds with observations from a study by Pachón-Peña et al. (2016) that investigated the association between the immunophenotypic profile of ADSCs and their plasticity in lean and obese individuals. The authors indicated that increased proliferation and migration of ADSCs were only evident in cells derived from lean individuals (following exposure to low oxygen tension conditions) and that differences in proliferation, migration, and differentiation capacity correlated with an altered immunophenotypic profile in ADSCs derived from obese individuals (Pachón-Peña et al., 2016). The present study demonstrated that IL6 seems to be a vital factor influencing the proliferation of ADSCs but is unlikely to be the only factor. Although the association between IL6 and cellular proliferation is known, to our knowledge this is the first study to demonstrate that this association occurs independently of dyslipidemia in a patient-based study. Immuno-depletion of IL6 from the patient-derived serum prior to its *ex vivo* use is, however, needed if such an association is to be confirmed.

The observed association between serum IL6 concentrations and ADSCs proliferation is consistent with previous studies highlighting the importance of IL6 in the maintenance and growth of mesenchymal stem cells (Pricola et al., 2009; van de Vyver et al., 2016). The mechanism of which is most likely related to the pleiotropic effects of IL6 capable of eliciting both a pro- and anti-inflammatory response depending on the environmental context (Cox et al., 2017). Classical IL6 signaling is mediated through the association of the protein-bound receptor complex (IL6 + IL6R) with the gp130 receptor subunit and elicits an anti-inflammatory protective effect (Rose-John, 2012, 2017). In contrast, higher concentrations of IL6 triggers trans-signaling, where the IL6R is cleaved by ADAM17 to generate a soluble IL6R (sIL6R), that enables binding of the classical protein-receptor complex to cells that do not express IL6R (Rose-John, 2012, 2017). The alternative signaling pathway therefore increases the target cell populations and by doing so amplifies the inflammatory response (Rose-John, 2012, 2017). This pleiotropic effect could potentially explain the observed dose dependent proliferative effect of IL6 on ADSCs. Some studies have, however, shown that IL6 alone has no proliferative effect on progenitor cells, instead it functions in synergy with multiple other cytokines such as OSM, LIF, SCF and GCSF (Wolfenden and Newman, 1992; Miura et al., 1993; Jacobsen et al., 1994; Lee et al., 2009) to promote cell growth. Disruption of systemic IL6 concentrations could therefore affect the proliferation of endogenous ADSCs.

The importance of the finely balanced cytokine expression profile in ADSC functional responses is supported by Strong et al. (2015). The authors indicated that specific levels of inflammatory cytokine induction are dependent on the stage and type of ADSC differentiation. Taken together, with our results, these studies indicate that long-term disruption to the systemic cytokine balance can be detrimental to the multifunctional properties of ADSCs. Dysregulation of endogenous ADSC function could in turn lead to metabolic disease progression in addition to compromising the therapeutic efficacy of these cells.

## Limitations and Future Perspectives

Even though, the conclusions from this study is very specific to a black African female population, it emphasizes that the metabolic and inflammatory profile of patients should be considered when evaluating the reliability of ADSCs for therapeutic use. Our study furthermore demonstrated that regardless of metabolic disease progression, the systemic concentrations of specifically IL6 influence the self-renewal (proliferation) of ADSCs. Although the association between IL6 and cellular proliferation is known, to our knowledge this is the first study to demonstrate that this association occurs independently of dyslipidemia in a patient-based study. The importance of this finding lies in the clinical translation of our research, suggesting that the low-grade inflammation in obese reproductive aged females could influence ADSC function and can potentially contribute to disease progression and the development of co-morbidities. This is evident not only in the association between IL6 and ADSC proliferation, but also in the decreased pool of circulating stem/progenitor cells despite elevated levels of the chemokine SDF1α.

In addition, this study demonstrated that although dyslipidemia did not seem to influence ADSC proliferation; the presence of an abnormal lipid profile in serum did result in the accumulation of lipid droplets during osteogenic differentiation. The cross-sectional nature and relatively small sample size are limitations of the current study. Future studies should investigate the influence of genetic variation during metabolic diseases and aim to identify novel factors that could influence ADSC functionality.

## Author Contributions

AS was responsible for participant recruitment, execution of research, and data collection and analysis. MMC performed all the DXA scans and was responsible for the body composition data analysis. MK assisted with serum analysis. WF and MC contributed to the interpretation of data and editing of the manuscript. MvdV was responsible for conceptual design of the study, data interpretation, analysis, writing, and editing of the manuscript.

## Conflict of Interest Statement

The authors declare that the research was conducted in the absence of any commercial or financial relationships that could be construed as a potential conflict of interest.

## Abbreviations

ADSCs: adipose derived stem cells
AM: adipogenic media
BMI: body mass index
C: healthy lean control group
cm: centimeter
CRP: C reactive protein
DMEM: Dulbecco’s modified Eagle medium
DXA: dual energy X-ray absorptiometry
FBG: fasting blood glucose
FBS: fetal bovine serum
FMO: fluorochrome minus one
GCSF: granulocyte colony stimulating factor
HDL: high density lipoprotein
HSA: human serum albumin
HSCs: hematopoietic stem cells
IFNγ: interferon gamma
IL: interleukin
IL6R: interleukin 6 receptor
kg: kilogram
LDL: low density lipoprotein
LIF: leukemia inhibitory factor
m: meter
MetS: metabolic syndrome group
OB: overweight/obese group
OM: osteogenic media
OSM: oncostatin M
PBMC: peripheral blood mononuclear cells
PBS: phosphate saline solution
RPM: revolutions per minute
SCF: stem cell factor
SDF1α: stromal derived factor 1 alpha
SGM: standard growth media
T2DM: type 2 diabetes mellitus
TF/LF: trunk fat-to-limb fat ratio
TGS: triglycerides
TNF: tumor necrosis factor
WHO: world health organization
WHR: waist-to-hip ratio.
